# Health Effects of Exposure to Environmental Pollutants: The Combination of Traditional and Emerging Pollutants

**DOI:** 10.3390/toxics13080641

**Published:** 2025-07-29

**Authors:** Zhangjian Chen, Guang Jia

**Affiliations:** 1 Department of Occupational and Environmental Health Sciences, School of Public Health, Peking University, Beijing 100191, China; 2 Beijing Key Laboratory of Toxicological Research and Risk Assessment for Food Safety, School of Public Health, Peking University, Beijing 100191, China

## 1. Introduction

This editorial introduces the Special Issue titled “Health Effects of Exposure to Environmental Pollutants”. The health effects of environmental pollutants, including traditional pollutants (such as particulate matters and heavy metals) and emerging pollutants (such as nanoparticles), has received widespread attention [1]. These pollutants can have serious impacts on the environment and human health. Research on the potential toxic effects and mechanisms of environmental pollutants can provide a scientific basis for related prevention work.

This Special Issue contains 11 articles: 10 research papers and 1 communication. The research papers focus on the potential toxicity and mechanisms of environmental pollutants affecting humans or the environment. Some papers highlight the potential toxicity and mechanisms of emerging pollutants such as nanomaterials using novel methods, such as multi-omics approaches. Some papers still focus on traditional pollutants, such as particulate matter, heavy metals, arsenic, benzene and its homologues, but they explore novel aspects in terms of toxicity mechanisms or biomarkers, such as microRNAs or circular RNAs. The only communication explored the associations between uranium and radium in groundwater and incidence of colorectal cancer in Georgia counties, USA.

## 2. An Overview of Published Articles

This Special Issue focuses on the potential toxic effects and mechanisms of both emerging and traditional environmental pollutants. Emerging pollutants are represented by nanoparticles. Shi et al. (Contribution 1) explore the size-dependent hepatotoxicity of nano-silica. Through in vitro cell experiments combined with multi-omics methods, the authors try to clarify the early health effects and key toxicity pathways of nanoparticles. Gao et al. (Contribution 2) focus on the potential respiratory toxicity of nano-zinc oxide, using animal experiments to study the MRI morphological effects on the olfactory epithelium and olfactory bulb of rats after intranasal instillation. There are many types of nanoparticles, and these two contributions are representative. They take typical nanoparticles as the research object, using in vitro cell experiments and in vivo animal experiments, respectively, combined with innovative technologies such as multi-omics, to elucidate their potential negative health effects and mechanisms. The research paradigm is worthy of reference for future studies.

In this Special Issue, traditional environmental pollutants are represented by air pollutants. Sun et al. (Contribution 3) investigate the effect of ambient air pollutants on the severity of allergic rhinitis symptoms. A prospective follow-up study was conducted among patients with allergic rhinitis, and they found an association between short-term exposure to air pollutants and exacerbation of nasal symptoms in patients with allergic rhinitis. Wang et al. (Contribution 4) elucidate the role of Glycoprotein A Repetition Predominant (GARP) protein in PM_2.5_-induced epithelial–mesenchymal transition (EMT) through the TGF-β/SMAD pathway in pulmonary epithelial cells and discuss the therapeutic potential of lentinan. Yang et al. (Contribution 5) focus on the neurotoxicity of PM_2.5_ and attempt to elucidate the related gut–brain axis mechanism using a multi-omics approach.

In real-world environments, human exposure is often not to a single pollutant but to a combination of multiple pollutants [2]. Therefore, studying the health effects and mechanisms of combined exposure to environmental pollutants is of great significance. Zhang et al. (Contribution 6) focus on various endocrine-disrupting chemicals (EDCs), including perchlorate, nitrate, and thiocyanate, exploring their association with female infertility and the mediation of obesity. Mascari et al. (Contribution 7) focus on the associations between environmental pollutant mixtures and red blood cell folate concentrations, finding that higher exposure to PFASs, metals, and PAHs is associated with lower red blood cell folate concentrations. Zheng et al. (Contribution 8) focus on the association between combined exposure to various heavy metals and the aging biomarker α-Klotho, finding that exposure to certain metals, particularly in combination, may reduce serum α-Klotho levels, potentially accelerating aging processes. Li et al. (Contribution 9) explore the effects of co-exposure to benzene, toluene, and xylene (BTX) on genetic damage, providing further support for the gene-environment interactions of BTX co-exposure and microRNA single-nucleotide substitutions (mirSNPs) in determining the severity of DNA strand breaks. Xu et al. (Contribution 10) also focus on the important role of non-coding RNA in toxic biomarkers and toxicity mechanisms, but unlike contribution 9, they focus on circular RNA. The authors found that Circ_0000284 is involved in arsenite-induced hepatic insulin resistance by blocking the plasma membrane translocation of GLUT4 in hepatocytes via IGF2BP2/PPAR-γ.

Rooney et al. (Contribution 11) contribute a communication exploring the carcinogenic effects of radioactive pollutants through an ecological study. The study found that radium in groundwater may be linked with an increased incidence of colorectal cancer (CRC).

## 3. Conclusions

The health effects of environmental pollutants require continuous attention, which serves as the foundation for establishing environmental standards and formulating environmental protection strategies. There is a wide range of environmental pollutants, and more research evidence is needed on the potential negative health effects and related mechanisms of traditional and emerging pollutants. The health effects of combined exposure to multiple pollutants on the human body are one of the important research topics in the field of environmental health. The environmental health effects are also diverse and complex, and new research methods, including high-throughput methods such as multi-omics, deserve attention.
